# Cost-effectiveness of ticagrelor versus clopidogrel for the prevention of atherothrombotic events in adult patients with acute coronary syndrome in Germany

**DOI:** 10.1007/s00392-013-0552-7

**Published:** 2013-03-09

**Authors:** Ulrike Theidel, Christian Asseburg, Evangelos Giannitsis, Hugo Katus

**Affiliations:** 1 Hannover, Germany; 2 Kuopio, Finland; 3 Heidelberg, Germany

**Keywords:** Cost-effectiveness, Ticagrelor, Acute coronary syndrome, Prevention, Long-term impact, Germany

## Abstract

The aim of this health economic analysis was to compare the cost-effectiveness of ticagrelor versus clopidogrel within the German health care system. A two-part decision model was adapted to compare treatment with ticagrelor or clopidogrel in a low-dose acetylsalicylic acid (ASA) cohort (≤150 mg) for all ACS patients and subtypes NSTEMI/IA and STEMI. A decision-tree approach was chosen for the first year after initial hospitalization based on trial observations from a subgroup of the PLATO study. Subsequent years were estimated by a Markov model. Following a macro-costing approach, costs were based on official tariffs and published literature. Extensive sensitivity analyses were performed to test the robustness of the model. One-year treatment with ticagrelor is associated with an estimated 0.1796 life-years gained (LYG) and gained 0.1570 quality-adjusted life-years (QALY), respectively, over the lifetime horizon. Overall average cost with ticagrelor is estimated to be EUR 11,815 vs. EUR 11,387 with generic clopidogrel over a lifetime horizon. The incremental cost-effectiveness ratio (ICER) was EUR 2,385 per LYG (EUR 2,728 per QALY). Comparing ticagrelor with Plavix^®^ or the lowest priced generic clopidogrel, ICER ranges from dominant to EUR 3,118 per LYG (EUR 3,567 per QALY). These findings are robust under various additional sensitivity analyses. Hence, 12 months of ACS treatment using ticagrelor/ASA instead of clopidogrel/ASA may offer a cost-effective therapeutic option, even when the generic price for clopidogrel is employed.

**Electronic supplementary material:**

The online version of this article (doi:10.1007/s00392-013-0552-7) contains supplementary material, which is available to authorized users.

## Introduction

In Germany, every year more than 400,000 patients are admitted to hospitals for suspected acute coronary syndrome (ACS) [1]. Cardiovascular (CV) disease is the leading cause of mortality in Germany, with more than 60,000 deaths due to acute or recurrent myocardial infarction [2]. Despite high resource use and services supplied to these patients mortality rates of 30 % or higher have been reported 1 year post-ACS [3–5]. Therefore, the reduction of CV event rates, particularly CV and all-cause mortality, still remains a key priority. Effective strategies to reduce CV mortality include reduction of pre-hospital and hospital delays, preferred use of an appropriately timed invasive strategy with percutaneous coronary intervention (PCI) and coronary stenting, use of more potent anti-platelet and anti-thrombin-inducing drugs, and comprehensive secondary prevention including utilization of acetylcholinesterase (ACE)-inhibitors, beta-blockers and statins. A 10 % increase of guideline adherence has been shown to reduce in-hospital mortality rates by 10 % [6] which makes this a desirable task.

Dual therapy with acetylsalicylic acid (ASA) and clopidogrel is a standard treatment option in patients with ACS. Treatment is recommended to start as early as possible and to be continued for 12 months post-ACS [7]. The efficacy of clopidogrel, a second generation thienopyridine that blocks the adenosine diphosphate (ADP) receptor on platelets is hampered by a slow and variable transformation of the prodrug to the active metabolite, modest and variable platelet inhibition, an increased risk of bleeding and an increased risk of stent thrombosis and myocardial infarction in patients with a poor response. Ticagrelor, a novel reversible and direct-acting oral antagonist of the adenosine diphosphate receptor P2Y12, showed faster, higher, and more consistent P2Y12 inhibition than clopidogrel. The pivotal PLATelet inhibition and patient outcomes (PLATO) phase III trial showed that ticagrelor was superior to clopidogrel for the prevention of CV death, myocardial infarction (MI), or stroke (9.8 vs. 11.7 % at 12 months; 16 % RRR; 95 % CI, 0.77–0.92; *p* < 0.001) without a significant increase of major bleeding (11.6 vs. 11.2 %, *p* = 0.43). The primary efficacy endpoint was driven by CV death (4.0 vs. 5.1 %, *p* = 0.001) and myocardial infarction (MI) (5.8 vs. 6.9 %, *p* = 0.005) with no difference in stroke (1.5 vs. 1.3 %, *p* = 0.22). Secondary safety endpoints show a significant increase in non-CABG-related spontaneous major bleedings (4.5 vs. 3.8 %, *p* = 0.03) and episodes of any dyspnea (13.8 vs. 7.8 %) and more bradycardic events (4.7 vs. 4.4 %) in a broad population of patients with ACS. There was no significant difference in the incidence of fatal bleedings (*p* = 0.66) [8].

In the PLATO study some patients received higher dosages of ASA, especially in centers outside the EU. In a pre-specified subgroup analysis, a significant interaction between treatment and region (*p* = 0.045) was shown [8]. In a treatment-by-region analysis Mahaffey et al. [9] quantified how much of the regional interaction could be explained by patient characteristics and concomitant treatments, including aspirin maintenance therapy. Adjusted analyses showed that ticagrelor was associated with better outcomes compared with clopidogrel in patients taking low-dose maintenance aspirin, with statistical superiority in the rest of the world and similar outcomes in the US cohort. Thus, the aspirin maintenance dose seems to offer a possible explanation for regional differences.

In Germany, the recommended dosage of ASA in combination with ticagrelor ranges from 75 up to 150 mg per day [10]. Addressing that issue and according to the requirements of the recently implemented benefit assessment regulation for new drugs (Arzneimittelmarktneuordnungsgesetz, AMNOG), a subgroup analysis was performed with the PLATO results evaluating the subset of patients in the study cohort receiving ≤150 mg ASA (ASA low-dose cohort). Data were presented as part of the benefit assessment of ticagrelor to the Federal Joint Committee (Gemeinsamer Bundesausschuss, G-BA) in Germany [11] and showed more favorable results than for the overall cohort: composite endpoint (7.9 vs. 10.2 % at 12 months; 22 % RRR; 95 % CI, 0.70–0.87; *p* < 0.0001), also driven by CV death (3.1 vs. 4.4 %; 29 % RRR; CI 95 %, 0.60–0.84; *p* < 0.0001) and MI (4.8 vs. 6.1 %, 21 % RRR; CI 95 %, 0.69–0.91, *p* = 0.0008). No differences in stroke were found (1.3 vs. 1.1 %; *p* = 0.2669). Secondary safety endpoints showed no significant increase in non-CABG-related spontaneous major bleedings (4.3 vs. 3.6 %, *p* = 0.06). Incidence of fatal bleedings also reached no significance (*p* = 0.99) (for more details see “Supplementary Material”).

The published benefit assessment for ticagrelor [12] reported an added clinical benefit for patients without ST-segment elevation (NSTEMI) and unstable angina (UA). The main inclusion criteria for patients with ST-segment elevation (STEMI) of the PLATO study was a planned PCI. In this population, the comparator for the benefit assessment was prasugrel according to the recommendation of G-BA. Based on an indirect comparison with prasugrel, the assessment of the Institute for Quality and Efficiency in Health Care (Institut für Qualität und Wirtschaftlichkeit im Gesundheitswesen, IQWiG) came to the conclusion that not enough evidence versus prasugrel could be presented by the dossier for these patients.

Aim of the present study was to assess the cost-effectiveness of ticagrelor over lifetime with a treatment period of 12 months compared to clopidogrel according to the requirements of the benefit assessment (ASA low-dose cohort). Cost-effectiveness was evaluated for ACS subtypes (NSTEMI/UA and STEMI) and, to get a complete overview for the ASA low-dose cohort, for all ACS patients.

## Methods

### Patients

PLATO (ClinicalTrials.gov identifier NCT00391872) was an international, prospective, randomized, double-blind, double dummy, event-driven trial in patients hospitalized for NSTEMI that was managed invasively or medically, or STEMI scheduled for primary PCI strategy. Details of the design, population, and outcome measures for the trial and for pre-specified subgroups have been published elsewhere [8]. Patients were randomized to receive either ticagrelor or clopidogrel within 24 h of onset of the most recent cardiac ischemic symptoms and before PCI. Ticagrelor-treated patients received a 180 mg loading dose followed by a maintenance dose of 90 mg BID. Clopidogrel-treated patients who had not already received a loading dose of open-label clopidogrel or who had not been taking clopidogrel or ticlopidine for >5 days before randomization received a 300-mg loading dose followed by 75 mg QD. The remaining patients received 75 mg clopidogrel as their first dose. Patients undergoing PCI received an additional 90 mg dose of ticagrelor/placebo at procedures >24 h after randomization and, at the discretion of the investigator, an additional 300 mg clopidogrel/placebo at any time relative to randomization. All patients received 75–100 mg/day acetylsalicylic acid unless intolerant. For patients not previously receiving ASA, a loading dose of 325 mg was preferred (although a dose of 160–500 mg was allowed). After stent placement, an ASA dose up to 325 mg/day was allowed for up to 6 months, and a lower dose was used thereafter. Outpatient visits were scheduled up to 12 months, with a safety follow-up visit 1 month after end of treatment. The randomized treatment was scheduled to continue for 12 months, but patients left the study at their 6- or 9-month visit if the targeted number of 1,780 primary endpoint events had occurred by that time.

### Cost-effectiveness study

Based on clinical data derived from the PLATO study, a two-part decision-analytic model, comprising a 1-year decision tree and a long-term Markov model, was adapted to estimate lifetime costs as well as health outcomes. The model structure was informed by earlier studies in this field. The main difference to the already existing multinational model [13, 14] is the used macro-costing approach to generate the cost data. The primary health outcomes are mean cost and life-years gained (LYG) of treating ACS patients for 1 year with ticagrelor plus ASA compared with clopidogrel plus ASA. In addition to LYs, quality-adjusted life-years (QALYs) are estimated in the model secondarily. Possible events in the model are “overall death”, “myocardial infarction”, and “stroke”. Adverse and subsequent events were not explicitly included in the analysis. Both items implicitly are still considered in the QALY analysis and via the inclusion of associated cost. Subgroup analysis was done for NSTEMI/UA and STEMI. After a non-fatal event, no further events were incorporated as these events occurred very seldom during the clinical trial.

The time horizon within a Markov model is always determined by the disease and the chosen perspective of evaluation. Even if the therapy of dual platelet inhibition is limited to the first 12 months after ACS index hospitalization, long-term consequences of a chronic disease will continue to have an impact throughout the remaining lifetime. As mentioned above, ACS is an event that changes the prognosis of a patient permanently for the entire life. Thus, in the base case the model is evaluated over lifetime and hence extrapolates beyond the study duration of the pivotal study. First year results were analyzed solely with a decision-tree approach, i.e., for the first year the study data of the phase III study was used.

**Fig. 1:**
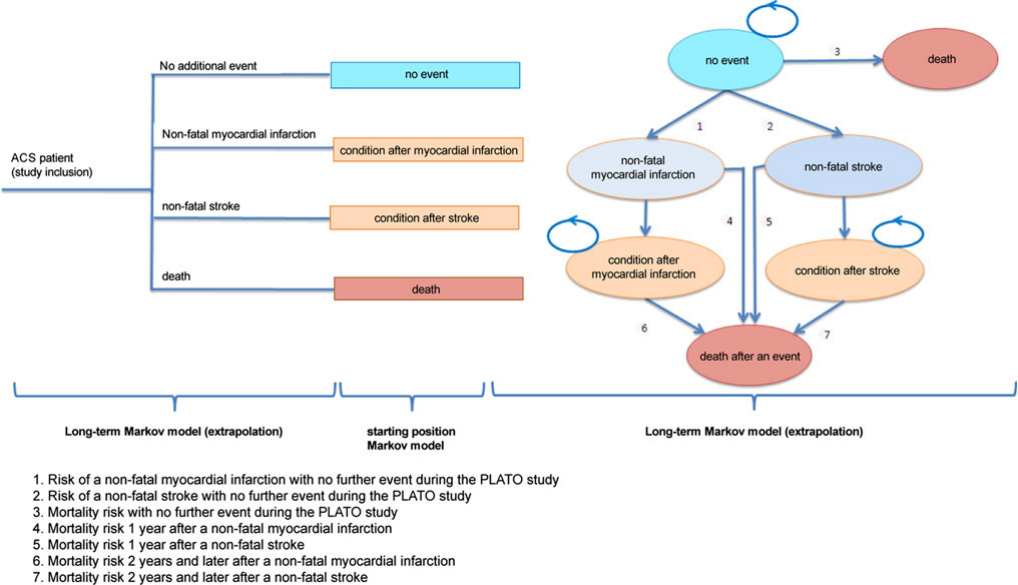
Model structure used for all subgroup analyses

Treatment with ticagrelor or clopidogrel is recommended only for 12 months. Therefore, the patients receive the drug therapy in both arms only in the first year. In the second step for the long-term analysis, a Markov model approach was chosen with a cycle length of 12 months. As no long-term data are available after the first year of treatment, the conservative assumption was made that no relevant differences exist regarding the efficacy between both alternatives. Starting the model with the Markov approach with the second year the only difference between both model arms arises from the different distribution of patients in the different Markov states after the first year. Moreover, transition probabilities of clinical events are assumed to be independent of treatment arm in the long-term model; Fig. 1 shows the model schematically.

The Markov health states correspond to the clinical endpoints in the PLATO study: overall mortality, myocardial infarction, and stroke. Patients who did not have an event during the first year will start in the Markov model in the state “no event”. These patients may suffer a fatal myocardial infarction (MI) or stroke in every subsequent year (arrow 3 in Fig. 1) and may also transit to a “non-fatal MI” (arrow 1) or “non-fatal stroke” (arrow 2). Annual probabilities for these transitions were estimated by extrapolating the Weibull regression models corresponding to the clopidogrel arm of the PLATO study to obtain the probability of events during year 2, conditional on no event in year 1. Based on a comparison of the predicted proportion of patients with events and the observed Kaplan–Meier estimates from the PLATO study, the Weibull model was found to provide the best fit with the clinical data. These transition probabilities were assumed to be constant beyond year 2 in both arms. Whenever a fatal event occurs, a patient passes to the absorbing state of “Death”. Mortality due to non-cardiovascular causes (also part of arrow 3) was estimated using the current German mortality tables, and is presumed to be known with certainty. The overall mortality was estimated conservatively and no extraction was made to exclude the mortality due to cardiovascular causes from the German standard mortality. Hence, in the base case mortality is overestimated.

Additional mortality risk due to non-fatal myocardial infarction or non-fatal stroke in the long-term model (arrows 4–7) and risk of other mortality (arrow 3) are parameterized by inflating mortality through hazard ratios (HR), parameterized using log-normal distributions. In the base case, assumptions regarding these hazard ratios relating to post-event mortality were made based on data from publications [15] and Federal Health Monitoring (Gesundheitsberichterstattung des Bundes) [16] (HR “no event”: 1/HR “non-fatal MI” first year: 1.6/HR “non-fatal MI” second and subsequent years: 1.4/HR “non-fatal stroke” first year: 3.23/HR “non-fatal stroke” second and subsequent years: 1.5). To compare the impact of different assumptions regarding these hazard ratios, hazards provided from the Global model were used in a sensitivity analysis (HR “no event”: 2/HR “non-fatal MI” first year: 6/HR “non-fatal MI” second and subsequent years: 3/HR “non-fatal stroke” first year: 7.43/HR “non-fatal stroke” second and subsequent years: 3) [14].

Several validation rounds were conducted, which included testing the model for internal validity and revising programming errors.

Various sensitivity analyses were carried out to test the robustness of the results. For the probabilistic sensitivity analysis of the clinical effects and quality of life parameters, 10,000 iterations were conducted. Clinical efficacy data for the first year were represented by Weibull regression models that summarize the effect size and temporal distributions of the PLATO study events and associated uncertainty. This approach automatically includes the consideration of any correlations between effect size and “base line”, and was chosen because it can best reflect any uncertainties and their relations to each other. Cost data are considered as known with certainty, no matter from which source they are derived. Robustness to cost assumptions was tested in univariate sensitivity analyses.

The primary endpoints of the cost-effectiveness model are absolute and incremental LYG, in relation to overall therapy costs. For the subgroups NSTEMI/UA and STEMI the same model structure and sensitivity analyses were used. Only clinical and cost data were modified (see the following section).

### Model inputs

Transition probabilities for the disease conditions are based on PLATO results. For each study outcome, a Weibull parametric survival regression was fitted to the patient-level data, a statistical approach that respects the trial randomization scheme. The published hazard ratio determined within the scope of the semi-parametric Cox proportional hazard model, cannot be used directly for the modeling approach. For subsequent years, no data were available. Therefore, conservative assumptions were made with respect to the occurrence probabilities. The residual mortality was estimated on the basis of current German mortality tables.

In terms of quality of life, the decision tree represents the data as collected in the PLATO study. The long-term model contains quality of life as an average value appropriate to the age and deductions which illustrate morbidities (MI and stroke). All data are presented in Tables 1, 2 and 3.

**Table 1:** Model input parameters (overall ACS patient population ≤150 mg ASA)

	Ticagrelor	Clopidogrel	Source
*Model parameters during the first year*			
Probability of the endpoint (mean value)			
Non-fatal myocardial infarction	0.041	0.049	Weibull regression
Non-fatal stroke	0.008	0.008	Weibull regression
Death	0.036	0.050	Weibull regression
Utility values			
No event	0.875	0.878	PLATO data
Non-fatal myocardial infarction	0.817	0.801	PLATO data
Non-fatal stroke	0.748	0.720	PLATO data
Death	0.259	0.249	PLATO data
*Model parameters Markov model*	*Common to both treatment arms*		
Annual probability of the endpoint (mean value)			
Non-fatal myocardial infarction	0.021		Extrapolation from Weibull regression
Non-fatal stroke	0.004		Extrapolation from Weibull regression
Fatal CV event	0.019		Extrapolation from Weibull regression
Observed utility in the PLATO trial			
No event (age 60–69)	0.877		PLATO data
No event (age 70–79)	0.838		PLATO data
No event (age 80+)	0.773		PLATO data
Utility decrements			
Year 1 after a stroke	0.143		PLATO data
Year 2+ after a stroke	0.143		PLATO data
Year 1 after a myocardial infarction	0.068		PLATO data
Year 2+ after a myocardial infarction	0.068		PLATO data

**Table 2:** Model input parameters (NSTEMI/UA ≤150 mg ASA)

	Ticagrelor	Clopidogrel	Source
*Model parameters during the first year*			
Probability of the endpoint (mean value)			
Non-fatal myocardial infarction	0.052	0.058	Weibull regression
Non-fatal stroke	0.008	0.009	Weibull regression
Death	0.038	0.050	Weibull regression
Utility values			
No event	0.864	0.863	PLATO data
Non-fatal myocardial infarction	0.794	0.777	PLATO data
Non-fatal stroke	0.736	0.677	PLATO data
Death	0.275	0.235	PLATO data
*Model parameters Markov model*	*Common to both treatment arms*		
Annual probability of the endpoint (mean value)			
Non-fatal myocardial infarction	0.024		Extrapolation from Weibull regression
Non-fatal stroke	0.004		Extrapolation from Weibull regression
Fatal CV event	0.023		Extrapolation from Weibull regression
Observed utility in the PLATO trial			
No event (age 60–69)	0.864		PLATO data
No event (age 70–79)	0.826		PLATO data
No event (age 80+)	0.762		PLATO data
Utility decrements			
Year 1 after a stroke	0.157		PLATO data
Year 2+ after a stroke	0.157		PLATO data
Year 1 after a myocardial infarction	0.078		PLATO data
Year 2+ after a myocardial infarction	0.078		PLATO data

**Table 3:** Model input parameters (STEMI ≤150 mg ASA)

	Ticagrelor	Clopidogrel	Source
*Model parameters during the first year*			
Probability of the endpoint (mean value)			
Non-fatal myocardial infarction	0.026	0.038	Weibull regression
Non-fatal stroke	0.008	0.007	Weibull regression
Death	0.032	0.046	Weibull regression
Utility values			
No event	0.891	0.899	PLATO data
Non-fatal myocardial infarction	0.879	0.855	PLATO data
Non-fatal stroke	0.763	0.833	PLATO data
Death	0.228	0.281	PLATO data
*Model parameters Markov model*	*Common to both treatment arms*		
Annual probability of the endpoint (mean value)			
Non-fatal myocardial infarction	0.016		Extrapolation from Weibull regression
Non-fatal stroke	0.003		Extrapolation from Weibull regression
Fatal CV event	0.015		Extrapolation from Weibull regression
Observed utility in the PLATO trial			
No event (age 60–69)	0.895		PLATO data
No event (age 70–79)	0.856		PLATO data
No event (age 80+)	0.789		PLATO data
Utility decrements			
Year 1 after a stroke	0.097		PLATO data
Year 2+ after a stroke	0.097		PLATO data
Year 1 after a myocardial infarction	0.028		PLATO data
Year 2+ after a myocardial infarction	0.028		PLATO data

The calculation of health expenses is a challenge as there is no detailed information on health costs for treatment of ACS in Germany available from official sources. A study by Taylor et al. [17] indicates that costs amounted to approximately EUR 3.3 billion in 2004. The Federal Health Monitoring [18] reported direct costs for acute and recurrent myocardial infarctions of around EUR 1.8 billion in 2008. Looking at the costs of ACS treatment per capita the statutory health insurance (Gesetzliche Krankenversicherung, GKV) states annual costs between EUR 8,280 and EUR 11,067 per patient in Germany. The majority of the expenses are due to hospitalization (ranging from 77 to 83 %). Furthermore, disability, invalidity, or premature death due to acute myocardial infarction result in a loss of 1.6 % of all work years in 2006 (i.e., 64,000 person-years) affecting predominantly men (approximately 87 %). Thus, the loss of 127,000 work years by ischemic heart diseases translates into a national economic loss to society of approx. EUR 4.3 billion [4]. Therefore and while the cost data in particular cannot be taken from multinational randomized controlled trials (RCTs), a macro-costing approach was chosen for this analysis to generate cost data from other sources [19].

The PLATO-associated health economic substudy [20] provides initial resource use and cost structures via a micro-costing approach, but it does not completely cover the resource use in the context or from the perspective of the German statutory health insurance [21].

Hence, a macro-costing approach was chosen and additional relevant cost data for events were identified from publicly accessible databases and the literature. Unit cost inputs were selected on the basis of best available evidence and are standardized (inflation-adjusted) to the year 2009. To evaluate the cost-effectiveness for both treatments, the focus of interest is on costs or savings occurring after the initiation of a chosen therapy. For this, in the underlying macro-costing approach costs for the index hospitalization were excluded from the analysis as these costs are covered by lump sum payment (diagnosis-related group, DRG) regardless of the initiation of any pharmacological treatment.

For acute hospitalization events, data from the official German DRG browser were used. In reality, depending on the disease history of a patient, existing co-morbidities and the specific kind, of event patients will be classified into different DRGs. Therefore, relevant DRGs were identified in the browser using the corresponding ICD-10 codes resulting in a weighted cost average per case observed in the PLATO data. Information for time after hospitalization was focused on the clinical pathways. Costs of cardiological rehabilitation were included, comprising outpatient as well as inpatient resources subsequent to hospital discharge, followed by visits to general practitioner (GP) or visits to a cardiologist and nursing care if needed. Indirect costs such as sick leave or early retirement were not incorporated into the base case model as these costs are not relevant from the perspective of the statutory health insurance. Costs for management of adverse events are covered by lump sum payment for in-hospital or outpatient treatment. Therefore, no extra costs for adverse events were included.

The costs for death were reported by federal statistics. An average amount of EUR 8,650 [22] was applied to the distribution of death due to cardiovascular or other causes as observed in PLATO. In the base case scenario for patients in the “no event”—state drug costs only and no other health-care costs were included in both arms. The costs of medication in the base case scenario were calculated using pharmacy retail prices (public prices) without any discounts. The daily therapy costs (DTC) for ticagrelor are EUR 2.90 (EUR 147.57 per 100 tablets) [23]. For clopidogrel, a DTC of EUR 0.72 (average generic price) was used in the base case as well as EUR 0.35 for lowest generic and EUR 2.38 for Plavix^®^ in the sensitivity analyses [23]. All prices were calculated excluding compulsory rebates [23] (Table 4).

**Table 4:** Cost parameters (overall ACS patient population ≤150 mg ASA)

	Myocardial infarction	Stroke
First year in EUR		
Acute hospitalization (incl. early rehabilitation)	4,226 [22]	9,791 [22]
Further hospitalization	2,601 [23]	1,063 [24]
Rehabilitation	1,757 [25]	1,610 [24]
Doctor’s visit/nursing care	975 [23]	2,462 [24]
Total costs for the first year	**9,558**	**14,925**
The following years in EUR (Markov model)		
Further hospitalization	2,008 [26]	4,336 [27]
Rehabilitation (admission)	439 [25]	
Doctor’s visit/nursing care	974 [23]	
Total costs in the following years	**3,421**	**4,336**

Bold values are used in the model

Subgroup-specific cost data could be generated for the MI state only. All other MI cost and cost for stroke and death were assumed to be equal (Table 5).

**Table 5:** Cost parameters for subgroups (only myocardial infarction)

	NSTEMI/UA	STEMI
First year in EUR		
Acute hospitalization (incl. early rehabilitation)	3,793 [22]	5,648 [22]

In addition, indirect costs (early retirement and work disability) as well as additional costs for no primary (study) event were incorporated in a sensitivity analysis: EUR 2,744 [30] for a myocardial infarction in the first year, EUR 4,417 [26, 30, 31] for a stroke in the first and EUR 4,336 [29] in the following years.

In accordance to current guideline (Hannoveraner Konsens [32]) all costs and benefit components were subject to a discount rate of 3 % in the base case scenario. Different discount rates were tested in the sensitivity analysis.

## Results

On the basis of the described model, it can be expected that the total average costs of therapy with ticagrelor over the entire remaining lifetime in the base case scenario will accrue to an average of EUR 11,815, as compared to EUR 11,387 with generic clopidogrel (average generic price). This leads to incremental costs of EUR 428. Driven by the data from the PLATO study [8], it is expected that 20 clinical events can be prevented per 1,000 ACS patients in the first year. Translated to the entire lifespan, this leads to 0.1796 years of LYG (0.1570 QALYs). The costs per life-year gained are, therefore, EUR 2,385 (EUR 2,728) in the base case scenario. For detailed results see Table 6.

**Table 6:** Detailed results of the base case scenario for all subgroups

	Ticagrelor	Clopidogrel	Incremental	ICER
Overall ACS patient population ≤150 mg ASA				
Costs in EUR	11,815	11,387	428	
Life-years	12.1471	11.9674	0.1796	2,385
QALYs	10.1349	9.9779	0.1570	2,728
NSTEMI/UA				
Costs in EUR	12,554	12,049	505	
Life-years	11.6438	11.4853	0.1585	3,184
QALYs	9.5356	9.3935	0.1421	3,552
STEMI				
Costs EUR	10,453	10,179	274	
Life-years	12.7890	12.5968	0.1922	1,426
QALYs	10.9953	10.8341	0.1613	1,700

These results are based on the conservative assumption that there is no incremental clinical benefit from ticagrelor vs. clopidogrel beyond the first year of treatment.

**Fig. 2:**
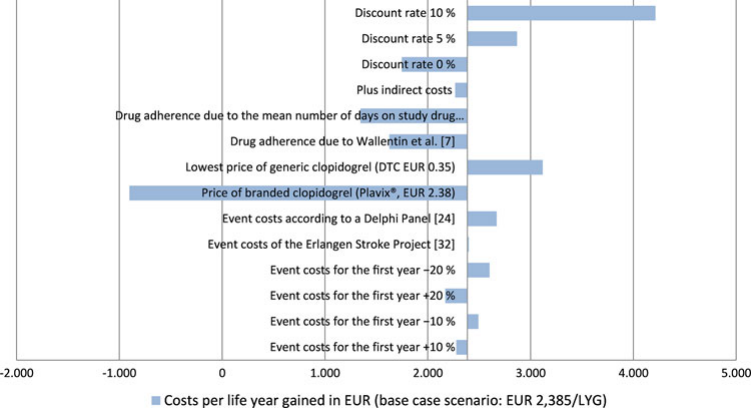
Results of univariate sensitivity analysis for overall ACS patient population ≤150 mg ASA

**Fig. 3:**
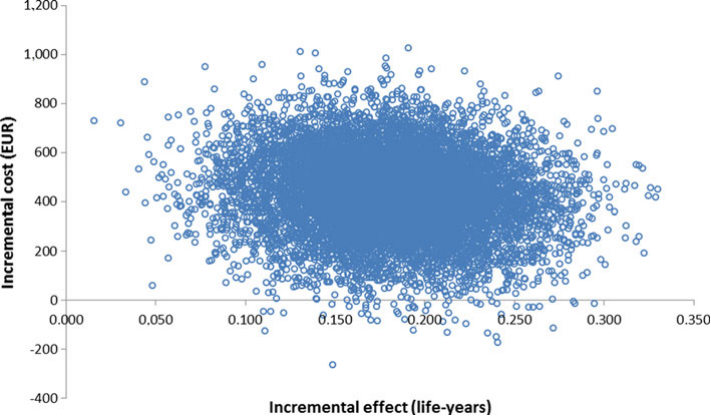
Results of the probabilistic sensitivity analysis

An overview of the results from the various scenarios explored with the model is provided in Fig. 2. The negative ICER shown for the sensitivity analysis regarding the price of branded clopidogrel is due to ticagrelor being cost-saving, i.e., dominating, in that sensitivity analysis. Sensitivity analysis for NSTEMI/UA and STEMI were not provided as the overall results have shown to be very stable (Fig. 3). The model has shown to be robust against changes in costs and clinical parameters. In particular, the variation in the price level of clopidogrel had a strong influence on the relative results. In order to evaluate the influence of generic substitution of clopidogrel hydrogenous sulfate with generic clopidogrel besilate and clopidogrel hydrochloride, a separate one-way sensitivity analysis used daily costs for clopidogrel of EUR 0.35. This resulted in incremental costs for ticagrelor of EUR 560 and an incremental cost-effectiveness ratio of EUR 3,118 per year of life gained. By contrast, ticagrelor becomes a dominant strategy when the branded price of clopidogrel is assumed.

**Fig. 4:**
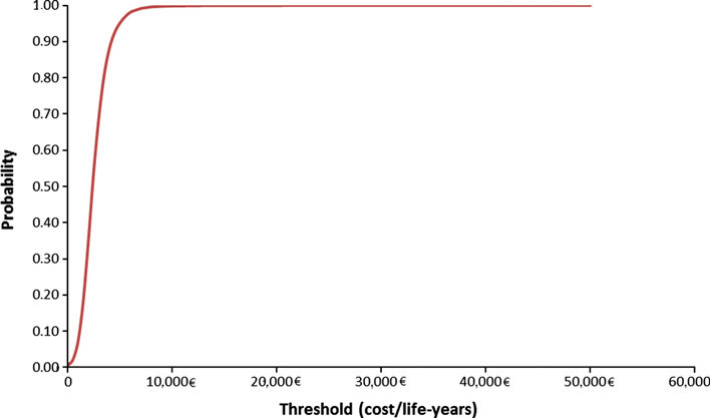
Cost-effectiveness acceptability curve

The results of the probabilistic sensitivity analysis are shown in Figs. 3 and 4.

## Discussion

The aim of this model was to evaluate the cost-effectiveness of a combination of ticagrelor and ASA instead of a combination of clopidogrel and ASA for guideline-recommended treatment over 12 months post-ACS in a low-dose ASA cohort for all ACS patients and subtypes NSTEMI/IA and STEMI. The results of the presented model are based on clinical events and health-related quality of life data from the ASA low-dose cohort of the PLATO study combined with mean cost from published literature and official tariffs like DRG. They are comparable to published results calculated for the total PLATO population. Nikolic et al. [14] reported findings that are in line with the cost-effectiveness of the present study. In that study, ACS patients treated with ticagrelor and ASA were projected to increase health-care costs of EUR 362 and gain a QALY of 0.13 compared with generic clopidogrel plus ASA. This yields a cost per QALY gained with ticagrelor of EUR 2,753. The cost per life-year gained was EUR 2,372. In addition, Theidel et al. [33] reported data, showing the cost-effectiveness for the treatment of ticagrelor in Germany based on overall PLATO results (not restricted to ASA low dose, no subgroups). In that study the incremental cost-effectiveness ratio (ICER) for the base case was EUR 3,274.

Due to the significant proportion of ASS on clinical effects, it might be expected that the results in this subgroup were inferior to the ASS group in total PLATO population. Insofar, our results substantiate the potential cost-effectiveness of ticagrelor over 12 months compared to clopidogrel in the country-specific German health care setting at the current price level—for patients receiving ticagrelor with ASA low dose and for subgroups NSTEMI/UA and STEMI. The model proves to be robust against changes in various sensitivity analyses. As seen with the PLATO health economics substudy, the results are mainly influenced by the commercial prize of clopidogrel that varies widely between the original drug compound (e.g., Plavix^®^) and the generic clopidogrel salts.

The cost-effectiveness of preventive treatments over short treatment periods is not always easy to determine, since treatment effects (here the prevention of events following an ACS) extend beyond the on-treatment period. Accordingly, the design should take into account clinical as well as monetary aspects over the entire remaining life span. One advantage of modeling is the opportunity to compile clinical evidence from different sources as well as data on the consumption of resources and costs of the respective health-care system [34]. The detailed results of the pivotal phase III study over a treatment period of 12 months were fully available for the presented model. For the subsequent years as a conservative assumption no relevant differences in efficacy were considered, disregarding any potential long-term benefits of ticagrelor. Cost parameters for Germany were taken from publicly accessible databases and literature. The influence of additional adverse events is only included via the main efficacy and benefit parameters as used in the PLATO trial.

Earlier clinical studies showed that there is a correlation between bleeding and mortality rates as well as recurrent myocardial infarctions. In a systematic review Cohen et al. [35] demonstrated that the impact of bleeding on mortality in ACS patients appears to be confined to the short term. Studies of long-term mortality consistently indicated that bleeding was not an independent predictor. However, in-hospital mortality seems to be strongly related to GRACE risk score in ACS patients, defined by age and systolic blood pressure [36]. Also Fitchett [37] concluded that there is no causal link between bleeding and increased coronary artery disease events (e.g., death) after an ACS episode. Nevertheless, in a retrospective chart review, Bufe et al. [38] found that bleeding could have an impact on morbidity and could therefore lead to longer in-hospital stays and to higher cost of hospitalization in German hospitals. By using the macro-costing approach, costs of bleeds and adverse events have already been included in the model. However, as no information on long-term sequelae is available this has not been added in the study.

In the literature, various approaches are being discussed regarding the generation of cost data for model adaptation [19]. The event costs of the presented model are based on various publications to ensure the best available evidence has been included. In this context, each hospitalization is depicted by the corresponding DRGs [24]. Data on consumption of resources during atherothrombotic events after hospital discharge have been provided by Brüggenjürgen et al. [25] in a Delphi panel. This study was first performed in 1997 and updated in 2004. All cost data that were used for the cost calculation were taken from publicly accessible documents and tariff catalogues. In terms of cardiological rehabilitation the publication of Zeidler et al. [27] a claims data analysis of the statutory health insurance data, was additionally used. Complete results from the Delphi panel were used in a sensitivity analysis. Cost data describing resource use for stroke care were alternatively taken from of a German health care services research study. Here, the authors explored in great detail the resource use of stroke patients (*n* = 558) over a period of 12 months yielding direct and indirect cost estimates [26]. In addition, with data from the Erlangen Stroke Registry [39] another data source was taken into consideration in a sensitivity analysis. Regarding the cost data for subsequent years, an extensive research of the literature was performed. Lamotte et al. [28] used data from Federal Statistics with regard to rehospitalization after myocardial infarction. Winter et al. [29] evaluated the long-term costs after a stroke in a highly detailed cost of illness study. Although that study is limited by its small sample size (*n* = 151), only minor deviations with respect to direct costs are suggested from the data of the Erlangen Stroke Registry. The cost of “death” has been calculated using DRG information and data from Federal Statistics. Since the therapy in PLATO was started following an ACS event with the aim to prevent MIs, strokes and associated death, no additional costs were incorporated for patients without additional events as observed in PLATO except, of course, for costs of medication. This assumption was tested in sensitivity analyses adding appropriate additional costs to determine the influence of these costs.

Observed utility values from the PLATO study are in the range of reported values for ischemic heart diseases measured by EQ-5D in different published studies. An evaluation of data from the MONICA registry in Germany [40] reports the quality of life several years after a myocardial infarction relative to the general population. Observed values in the registry population were similar to those observed in PLATO. No specific data for quality of life in German ACS population exist. A recently published review showed that published values are heterogenic, but the EQ-5D seems to be an appropriate questionnaire to measure the quality of life in cardiovascular diseases. Overall response to the questionnaire in the PLATO study was <70 % of the ITT population. There were no significant differences in quality of life between ticagrelor and clopidogrel reported from the trial [41, 42].

The main limiting factor of the model is the restriction to the PLATO data as its main source on treatment effects, and it shares the limitations of this trial, e.g., regarding specific subgroups and the duration of the recommended therapy. Our analysis may not apply to patients who were excluded from the trial. Beyond the PLATO trial, no real-world evidence could be generated. Duration of treatment and observed effect data are limited to the first year. Modeling the costs and health outcomes for subsequent years is based on assumptions. Mortality rates during the acute ACS phase will most likely continue to decline in the future, but the prevalence of patients with a prior MI and stable coronary artery disease will continue to increase. [43] To be able to distinguish different ACS risk groups and assign the appropriate long-term mortality, future model revisions should also try to estimate the cost-effectiveness of ticagrelor versus clopidogrel separately for other subgroups [44, 45].

To simplify the model, no subsequent events, therapy switches or various treatment durations were taken into consideration. These could, however, be relevant in clinical practice.

Furthermore, all cost data are based on literature research or official databases (e.g., DRG Browser) with the same mean annual cost per health state regardless of initial intervention. To obtain more detailed data regarding resource use of each treatment strategy and associated cost or benefits, a claims data analysis could support the model in the future with data from a real-world setting, when ticagrelor has a greater market penetration. It would also give the opportunity to observe changes in clinical practice.

This model was set up specifically to evaluate the German context. Even in other countries with similar health-care systems, the results of this study may not apply. When interpreting the implications of this study, the reader is advised to keep in mind that this is a modeling study that combines PLATO data and assumptions on long-term outcomes that are reasonable in the absence of hard data. In a decision context, the uncertainty related to these modeling assumptions must be balanced against the possibility of substantiating the model with actual data obtained in the German setting and over a longer time horizon.

There are no published willingness-to-pay threshold values for the cost-effectiveness of therapy in Germany. While no universal threshold for cost-effectiveness exists, a cost per additional life-year gained or quality-adjusted life-year (QALY) in the range of EUR 25,000 (USD 33,000) to EUR 38,000 (USD 50,000) is generally considered as cost-effective [46, 47]. Applying this generally accepted benchmark, treatment with ticagrelor would be considered a cost-effective option in Germany with costs per life-year gained of EUR 2,385 in the base case scenario and EUR 3,118 per life-year gained when less expensive generic clopidogrel compounds are being used. With a presumed QALY threshold of EUR 25,000/EUR 38,000 the probability of being cost-effective would be 99.98 %/99.99 % for the overall ACS population, 99.24 %/99.50 % for NSTEMI/UA, and 99.57 %/99.66 % for STEMI, respectively. But results may not fully capture the German setting because not all required inputs were available by publicly accessible literature and databases.

In conclusion, treatment of ACS with ticagrelor instead of clopidogrel over a time period of 12 months should offer a cost-effective therapeutic option in the context of the German health care system, even when considering the lower cost of generic clopidogrel. In addition, our findings are consistent with the cost-effectiveness of ticagrelor as seen in the international PLATO substudy.

## Electronic supplementary material

The following data are from the additional analysis of the benefit dossier [11, 12, 48].

Below is the link to the electronic supplementary material.

Table 7: Results of major efficacy endpoints (Overall ACS patient population ≤ 150 mg ASA) (DOCX 17 kb)

Table 8: Results of major efficacy endpoints (NSTEMI/UA ≤ 150 mg ASA) (DOCX 17 kb)

Table 9: Results of major efficacy endpoints (STEMI ≤ 150 mg ASA) (DOCX 17 kb)

Table 10: Results of major safety endpoints (Overall ACS patient population ≤ 150 mg ASA) (DOCX 25 kb)

Table 11: Results of major safety endpoints (NSTEMI/UA ≤ 150 mg ASA) (DOCX 25 kb)

Table 12: Results of major safety endpoints (STEMI ≤ 150 mg ASA) (DOCX 25 kb)
