# Supplementary material for: Cost-effectiveness of ticagrelor versus clopidogrel for the prevention of atherothrombotic events in adult patients with acute coronary syndrome in Germany
Source: Clin Res Cardiol. 2013 Mar 9;102(6):447–58. doi: 10.1007/s00392-013-0552-7 (PMC4269206; doi:10.1007/s00392-013-0552-7)
Supplement: Supplementary file 1 — Table 7: Results of major efficacy endpoints (Overall ACS patient population ≤ 150 mg ASA) (DOCX 17 kb) [file 392_2013_552_MOESM1_ESM.docx]

Table 7: Results of major efficacy endpoints (Overall ACS patient population ≤150 mg ASA)

| Endpoint | Ticagrelor + ASS | | Clopidogrel + ASS | | Ticagrelor vs. Clopidogrel | |
| --- | --- | --- | --- | --- | --- | --- |
|  | N | n (KM %) | N | n (KM %) | Hazard Ratio (95 %-KI) | p-Value |
| Composite of CV Death/MI (excl. silent MI)/Stroke | 8,025 | 594 (7.9 %) | 8,034 | 759 (10.2 %) | 0.78 (0.70-0.87) | <0.0001 |
| MI (excl. silent MI) | 8,025 | 360 (4.8 %) | 8,034 | 453 (6.1 %) | 0.79 (0.69-0.91) | 0.0008 |
| CV Death | 8,025 | 231 (3.1 %) | 8,034 | 325 (4.4 %) | 0.71 (0.60-0.84) | <0.0001 |
| Stroke | 8,025 | 97 (1.3 %) | 8,034 | 82 (1.1 %) | 1.18 (0.88-1.58) | 0.2669 |
| Death from any cause | 8,025 | 269 (3.6 %) | 8,034 | 376 (5.1 %) | 0.71 (0.61-0.84) | <0.0001 |
| Severe recurrent ischemia | 8,025 | 257 (3.4 %) | 8,034 | 301 (4.0 %) | 0.85 (0.72-1.00) | 0.0571 |
| Rehospitalization due to cardiovascular causes | 8,025 | 570 (7.8 %) | 8,034 | 613 (8.5 %) | 0.93 (0.83-1.04) | 0.1886 |
