# Supplementary material for: Cost-effectiveness of ticagrelor versus clopidogrel for the prevention of atherothrombotic events in adult patients with acute coronary syndrome in Germany
Source: Clin Res Cardiol. 2013 Mar 9;102(6):447–58. doi: 10.1007/s00392-013-0552-7 (PMC4269206; doi:10.1007/s00392-013-0552-7)
Supplement: Supplementary file 2 — Table 8: Results of major efficacy endpoints (NSTEMI/UA ≤ 150 mg ASA) (DOCX 17 kb) [file 392_2013_552_MOESM2_ESM.docx]

Table 8: Results of major efficacy endpoints (NSTEMI/UA ≤150 mg ASA)

| Endpoint | Ticagrelor + ASS | | Clopidogrel + ASS | | Ticagrelor vs. Clopidogrel | |
| --- | --- | --- | --- | --- | --- | --- |
|  | N | n (KM %) | N | n (KM %) | Hazard Ratio (95 %-KI) | p-Value |
| Composite of CV Death/MI (excl. silent MI)/Stroke | 4,725 | 397 (8.9 %) | 4,751 | 491 (11.2 %) | 0.81 (0.71-0.92) | 0.0015 |
| MI (excl. silent MI) | 4,725 | 260 (5.9 %) | 4,751 | 307 (7.0 %) | 0.85 (0.72-1.00) | 0.0473 |
| CV Death | 4,725 | 137 (3.1 %) | 4,751 | 197 (4.6 %) | 0.70 (0.56-0.87) | 0.0012 |
| Stroke | 4,725 | 58 (1.3 %) | 4,751 | 58 (1.4 %) | 1.00 (0.70-1.44) | 0.9914 |
| Death from any cause | 4,725 | 165 (3.8 %) | 4,751 | 226 (5.3 %) | 0.73 (0.60-0.89) | 0.0022 |
| Severe recurrent ischemia | 4,725 | 180 (4.0 %) | 4,751 | 198 (4.5 %) | 0.91 (0.74-1.11) | 0.3567 |
| Rehospitalization due to cardiovascular causes | 4,725 | 401 (9.2 %) | 4,751 | 418 (9.8 %) | 0.96 (0.84-1.10) | 0.5779 |
