# Supplementary material for: Cost-effectiveness of ticagrelor versus clopidogrel for the prevention of atherothrombotic events in adult patients with acute coronary syndrome in Germany
Source: Clin Res Cardiol. 2013 Mar 9;102(6):447–58. doi: 10.1007/s00392-013-0552-7 (PMC4269206; doi:10.1007/s00392-013-0552-7)
Supplement: Supplementary file 3 — Table 9: Results of major efficacy endpoints (STEMI ≤ 150 mg ASA) (DOCX 17 kb) [file 392_2013_552_MOESM3_ESM.docx]

Table 9: Results of major efficacy endpoints (STEMI ≤150 mg ASA)

| Endpoint | Ticagrelor + ASS | | Clopidogrel + ASS | | Ticagrelor vs. Clopidogrel | |
| --- | --- | --- | --- | --- | --- | --- |
|  | N | n (KM %) | N | n (KM %) | Hazard Ratio (95 %-KI) | p-Value |
| Composite of CV Death/MI (excl. silent MI)/Stroke | 3,145 | 182 (6.2 %) | 3,162 | 252 (8.5 %) | 0.72 (0.60-0.87) | 0.0007 |
| MI (excl. silent MI) | 3,145 | 96 (3.3 %) | 3,162 | 140 (4.8 %) | 0.68 (0.53-0.89) | 0.004 |
| CV Death | 3,145 | 84 (2.8 %) | 3,162 | 116 (3.9 %) | 0.73 (0.55-0.96) | 0.0260 |
| Stroke | 3,145 | 36 (1.3 %) | 3,162 | 24 (0.8 %) | 1.51 (0.90-2.53) | 0.1181 |
| Death from any cause | 3,145 | 93 (3.1 %) | 3,162 | 135 (4.5 %) | 0.69 (0.53-0.90) | 0.0062 |
| Severe recurrent ischemia | 3,145 | 75 (2.6 %) | 3,162 | 95 (3.2 %) | 0.79 (0.58-1.07) | 0.1275 |
| Rehospitalization due to cardiovascular causes | 3,145 | 161 (5.8 %) | 3,162 | 187 (6.5 %) | 0.86 (0.70-1.06) | 0.1603 |
