# Supplementary material for: Cost-effectiveness of ticagrelor versus clopidogrel for the prevention of atherothrombotic events in adult patients with acute coronary syndrome in Germany
Source: Clin Res Cardiol. 2013 Mar 9;102(6):447–58. doi: 10.1007/s00392-013-0552-7 (PMC4269206; doi:10.1007/s00392-013-0552-7)
Supplement: Supplementary file 4 — Table 10: Results of major safety endpoints (Overall ACS patient population ≤ 150 mg ASA) (DOCX 25 kb) [file 392_2013_552_MOESM4_ESM.docx]

Table 10: Results of major safety endpoints (Overall ACS patient population ≤150 mg ASA)

| Endpoint | Ticagrelor + ASS | | Clopidogrel + ASS | | Ticagrelor vs. Clopidogrel | |
| --- | --- | --- | --- | --- | --- | --- |
|  | N | n (KM %) | N | n (KM %) | Hazard Ratio (95 %-KI) | p-Value |
| Major bleeding  (study criteria) | 8,025 | 802 (11.0 %) | 8,034 | 773 (10.6 %) | 1.04 (0.94-1.15) | 0.4155 |
| Non-CABG related major bleeding  (study criteria) | 8,025 | 305 (4.3 %) | 8,034 | 261 (3.6 %) | 1.18 (1.00-1.39) | 0.0549 |
| CABG related major bleeding  (study criteria) | 8,025 | 513 (7.0 %) | 8,034 | 536 (7.4 %) | 0.96 (0.85-1.08) | 0.4918 |
| Life-threatening or fatal bleeding  (study criteria) | 8,025 | 398 (5.4 %) | 8,034 | 390 (5.3 %) | 1.03 (0.89-1.18) | 0.7221 |
| Fatal bleeding | 8,025 | 17 (0.3 %) | 8,034 | 17 (0.2 %) | 1.01 (0.51-1.97) | 0.9880 |
| Major or minor bleeding  (study criteria) | 8,025 | 1,131 (15.5 %) | 8,034 | 1,027 (14.0 %) | 1.11 (1.02-1.21) | 0.0141 |
| Non-CABG related major or minor bleeding  (study criteria) | 8,025 | 609 (8.4 %) | 8,034 | 491 (6.8 %) | 1.25 (1.11-1.41) | 0.0002 |
| CABG related major or minor bleeding  (study criteria) | 8,025 | 554 (7.6 %) | 8,034 | 588 (8.1 %) | 0.94 (0.84-1.06) | 0.3159 |
| Adverse events, any | 8,025 | 5,834 (72.7 %) | 8,034 | 5,554 (69.1 %) |  | <0.001 |
| Discontinuation of the study drug due to adverse events | 8,025 | 551 (6.9 %) | 8,034 | 408 (5.1 %) |  | <0.001 |
| Severe adverse events. any | 8,025 | 1,574 (19.6 %) | 8,034 | 1,584 (19.7 %) |  | 0.87 |
| Neoplasm arising during treatment, any | 8,025 | 118 (1.5 %) | 8,034 | 128 (1.6 %) |  | 0.56 |
| Neoplasm arising during treatment, malignant | 8,025 | 102 (1.3 %) | 8,034 | 102 (1.3 %) |  | 1.00 |
| Neoplasm arising during treatment, benign | 8,025 | 17 (0.2 %) | 8,034 | 27 (0.3 %) |  | 0.17 |
| Dyspnoe | 8,025 | 1,087 (13.6 %) | 8,034 | 632 (7.9 %) |  | <0.001 |
| Discontinuation of study treatment  due to dyspnoe | 8,025 | 67 (0.8 %) | 8,034 | 13 (0.2 %) |  | <0.001 |
| Pacemaker insertion | 8,025 | 70 (0.9 %) | 8,034 | 63 (0.8 %) |  | 0.54 |
| Syncope | 8,025 | 93 (1.2 %) | 8,034 | 66 (0.8 %) |  | 0.03 |
| Bradycardia | 8,025 | 362 (4.5 %) | 8,034 | 330 (4.1 %) |  | 0.21 |
| Heart block | 8,025 | 60 (0.8 %) | 8,034 | 56 (0.7 %) |  | 0.71 |
| Increase in serum uric acid from baseline value | | | | | | |
| At 1 month |  | 14±47 |  | 6±38 |  | < 0.001 |
| At 12 months |  | 15±53 |  | 7±32 |  | < 0.001 |
| 1 month after end of treatment |  | 7±45 |  | 8±40 |  | 0.96 |
| Increase in serum creatinine from baseline value | | | | | | |
| At 1 month |  | 10±21 |  | 8±21 |  | 0.003 |
| At 12 months |  | 11±22 |  | 9±22 |  | 0.002 |
| 1 month after end of treatment |  | 10±22 |  | 10±22 |  | 0.84 |
| Ventricular pauses | | | | | | |
| First week |  |  |  |  |  |  |
| ≥3 sec | 1,248 | 72 (5.8 %) | 1,220 | 44 (3.6 %) |  | 0.01 |
| ≥5 sec | 1,248 | 24 (1.9 %) | 1,220 | 15 (1.2 %) |  | 0.20 |
| At 30 days |  |  |  |  |  |  |
| ≥3 sec | 866 | 17 (2.0 %) | 879 | 14 (1.6 %) |  | 0.59 |
| ≥5 sec | 866 | 6 (0.7 %) | 879 | 5 (0.6 %) |  | 0.77 |
