# Supplementary material for: Cost-effectiveness of ticagrelor versus clopidogrel for the prevention of atherothrombotic events in adult patients with acute coronary syndrome in Germany
Source: Clin Res Cardiol. 2013 Mar 9;102(6):447–58. doi: 10.1007/s00392-013-0552-7 (PMC4269206; doi:10.1007/s00392-013-0552-7)
Supplement: Supplementary file 5 — Table 11: Results of major safety endpoints (NSTEMI/UA ≤ 150 mg ASA) (DOCX 25 kb) [file 392_2013_552_MOESM5_ESM.docx]

Table 11: Results of major safety endpoints (NSTEMI/UA ≤150 mg ASA)

| Endpoint | Ticagrelor + ASS | | Clopidogrel + ASS | | Ticagrelor vs. Clopidogrel | | | |  |
| --- | --- | --- | --- | --- | --- | --- | --- | --- | --- |
|  | N | n (KM %) | N | n (KM %) | Hazard Ratio (95 %-KI) | | p-Value | |  |
| Major bleeding  (study criteria) | 4,725 | 558 (13.0 %) | 4,751 | 529 (12.3 %) | 1.07 (0.95-1.20) | | 0.2742 | |  |
| Non-CABG related major bleeding  (study criteria) | 4,725 | 188 (4.5 %) | 4,751 | 160 (3.9 %) | 1.19 (0.97-1.48) | | 0.0981 | |  |
| CABG related major bleeding  (study criteria) | 4,725 | 383 (8.9 %) | 4,751 | 386 (8.9 %) | 1.00 (0.87-1.15) | | 0.9863 | |  |
| Life-threatening or fatal bleeding  (study criteria) | 4,725 | 285 (6.6 %) | 4,751 | 264 (6.2 %) | 1.09 (0.93-1.29) | | 0.2891 | |  |
| Fatal bleeding | 4,725 | 11 (0.3 %) | 4,751 | 14 (0.4 %) | 0.80 (0.36-1.76) | | 0.5742 | |  |
| Major or minor bleeding  (study criteria) | 4,725 | 767 (17.9 %) | 4,751 | 693 (16.1 %) | 1.13 (1.02-1.25) | | 0.0219 | |  |
| Non-CABG related major or minor bleeding (study criteria) | 4,725 | 385 (9.2 %) | 4,751 | 310 (7.4 %) | 1.27 (1.09-1.47) | | 0.0018 | |  |
| CABG related major or minor bleeding  (study criteria) | 4,725 | 407 (9.4 %) | 4,751 | 424 (9.7 %) | 0.97 (0.84-1.11) | | 0.6227 | |  |
| Adverse events, any | 4,725 | 3,418 (72.3 %) | 4,751 | 3,258 (68.6 %) |  | | <0.0001 | |  |
| Discontinuation of the study drug  due to adverse events | 4,725 | 389 (8.2 %) | 4,751 | 273 (5.7 %) |  | | <0.0001 | |  |
| Severe adverse events, any | 4,725 | 929 (19.7 %) | 4,751 | 941 (19.8 %) |  | | 0.88 | |  |
| Neoplasm arising during treatment, any | 4,725 | 69 (1.5 %) | 4,751 | 77 (1.6 %) |  | | 0.56 | |  |
| Neoplasm arising during treatment, malignant | 4,725 | 61 (1.3 %) | 4,751 | 62 (1.3 %) |  | | >0.9999 | |  |
| Neoplasm arising during treatment, benign | 4,725 | 9 (0.2 %) | 4,751 | 16 (0.3 %) |  | | 0.23 | |  |
| Dyspnoe | 4,725 | 662 (14.0 %) | 4,751 | 368 (7.7 %) |  | | <0.0001 | |  |
| Discontinuation of study treatment  due to dyspnoe | 4,725 | 54 (1.1 %) | 4,751 | 8 (0.2 %) |  | | <0.0001 | |  |
| Pacemaker insertion | 4,725 | 26 (0.6 %) | 4,751 | 33 (0.7 %) |  | | 0.43 | |  |
| Syncope | 4,725 | 60 (1.3 %) | 4,751 | 35 (0.7 %) |  | | 0.01 | |  |
| Bradycardia | 4,725 | 206 (4.4 %) | 4,751 | 164 (3.5 %) |  | | 0.02 | |  |
| Heart block | 4,725 | 25 (0.5 %) | 4,751 | 28 (0.6 %) |  | | 0.78 | |  |
| Increase in serum uric acid from baseline value | | | | | | | | |  |
| At 1 month |  | 13 ± 56 |  | 5 ± 34 | |  | | <.001 | |
| At 12 months |  | 15 ± 63 |  | 7 ± 34 | |  | | <.001 | |
| 1 month after end of treatment |  | 7 ± 53 |  | 7 ± 42 | |  | | 0.88 | |
| Increase in serum creatinine from baseline value | | | | | | | | |  |
| At 1 month |  | 9 ± 22 |  | 7 ± 19 | |  | | 0.01 | |
| At 12 months |  | 10 ± 22 |  | 9 ± 22 | |  | | 0.01 | |
| 1 month after end of treatment |  | 9 ± 22 |  | 9 ± 21 | |  | | 0.90 | |
| Ventricular pauses | | | | | | | | |  |
| First week |  |  |  |  | |  | |  | |
| ≥3 sec | 822 | 51 (6.2 %) | 823 | 29 (3.5 %) | |  | | 0.01 | |
| ≥5 sec | 822 | 18 (2.2 %) | 823 | 9 (1.1 %) | |  | | 0.08 | |
| At 30 days |  |  |  |  | |  | |  | |
| ≥3 sec | 567 | 14 (2.5 %) | 616 | 11 (1.8 %) | |  | | 0.43 | |
| ≥5 sec | 567 | 6 (1.1 %) | 616 | 4 (0.6 %) | |  | | 0.53 | |
