# Supplementary material for: Cost-effectiveness of ticagrelor versus clopidogrel for the prevention of atherothrombotic events in adult patients with acute coronary syndrome in Germany
Source: Clin Res Cardiol. 2013 Mar 9;102(6):447–58. doi: 10.1007/s00392-013-0552-7 (PMC4269206; doi:10.1007/s00392-013-0552-7)
Supplement: Supplementary file 6 — Table 12: Results of major safety endpoints (STEMI ≤ 150 mg ASA) (DOCX 25 kb) [file 392_2013_552_MOESM6_ESM.docx]

Table 12: Results of major safety endpoints (STEMI ≤150 mg ASA)

| Endpoint | Ticagrelor + ASS | | | | Clopidogrel + ASS | | | | | | | Ticagrelor vs. Clopidogrel | | |
| --- | --- | --- | --- | --- | --- | --- | --- | --- | --- | --- | --- | --- | --- | --- |
|  | N | n (KM %) | | | N | | | n (KM %) | | | | Hazard Ratio (95 %-KI) | p-Value | |
| Major bleeding  (study criteria) | 3,145 | 233 (8.1 %) | | | 3,162 | | | 229 (7.9 %) | | | | 1.02 (0.85-1.22) | 0.8637 | |
| Non-CABG related major bleeding  (study criteria) | 3,145 | 113 (3.9 %) | | | 3,162 | | | 95 (3.2 %) | | | | 1.19 (0.91-1.57) | 0.2022 | |
| CABG related major bleeding  (study criteria) | 3,145 | 123 (4.4 %) | | | 3,162 | | | 141 (5.0 %) | | | | 0.87 (0.68-1.10) | 0.2443 | |
| Life-threatening or fatal bleeding  (study criteria) | 3,145 | 108 (3.7 %) | | | 3,162 | | | 116 (4.0 %) | | | | 0.93 (0.72-1.21) | 0.5880 | |
| Fatal bleeding | 3,145 | 6 (0.2 %) | | | 3,162 | | | 3 (0.1 %) | | | | n.b. | n.b. | |
| Major or minor bleeding  (study criteria) | 3,145 | 351 (12.2 %) | | | 3,162 | | | 316 (10.8 %) | | | | 1.12 (0.96-1.30) | 0.1599 | |
| Non-CABG related major or minor bleeding  (study criteria) | 3,145 | 219 (7.5 %) | | | 3,162 | | | 172 (5.7 %) | | | | 1.28 (1.05-1.57) | 0.0145 | |
| CABG related major or minor bleeding  (study criteria) | 3,145 | 139 (5.0 %) | | | 3,162 | | | 155 (5.5 %) | | | | 0.89 (0.71-1.12) | 0.3198 | |
| Adverse events, any | 3,145 | 2,303 (73.2 %) | | | 3,162 | | | 2,206 (69.8 %) | | | |  | 0.002 | |
| Discontinuation of the study drug  due to adverse events | 3,145 | 150 (4.8 %) | | | 3,162 | | | 124 (3.9 %) | | | |  | 0.108 | |
| Severe adverse events, any | 3,145 | 624 (19.8 %) | | | 3,162 | | | 615 (19.4 %) | | | |  | 0.704 | |
| Neoplasm arising during treatment, any | 3,145 | 48 (1.5 %) | | | 3,162 | | | 51 (1.6 %) | | | |  | 0.84 | |
| Neoplasm arising during treatment, malignant | 3,145 | 41 (1.3 %) | | | 3,162 | | | 40 (1.3 %) | | | |  | 0.91 | |
| Neoplasm arising during treatment, benign | 3,145 | 7 (0.2 %) | | | 3,162 | | | 11 (0.3 %) | | | |  | 0.48 | |
| Dyspnoe | 3,145 | 401 (12.9 %) | | | 3,162 | | | 253 (8.0 %) | | | |  | <0.0001 | |
| Discontinuation of study treatment  due to dyspnoe | 3,145 | 13 (0.4 %) | | | 3,162 | | | 4 (0.1 %) | | | |  | 0.03 | |
| Pacemaker insertion | 3,145 | 43 (1.4 %) | | | 3,162 | | | 30 (0.9 %) | | | |  | 0.13 | |
| Syncope | 3,145 | 32 (1.0 %) | | | 3,162 | | | 29 (0.9 %) | | | |  | 0.70 | |
| Bradycardia | 3,145 | 155 (4.9 %) | | | 3,162 | | | 164 (5.2 %) | | | |  | 0.65 | |
| Heart block | 3,145 | 33 (1.0 %) | | | 3,162 | | | 28 (0.9 %) | | | |  | 0.52 | |
| Increase in serum uric acid from baseline value | | | | | | | | | | | | | | |
| At 1 month |  | | 16 ± 30 | | |  | | | 9 ± 43 | |  | | <0.0001 | |
| At 12 months |  | | 16 ± 35 | | |  | | | 8 ± 29 | |  | | <0.0001 | |
| 1 month after end of treatment |  | | 9± 31 | | |  | | | 9± 36 | |  | | 0.88 | |
| Increase in serum creatinine from baseline value | | | | | | | | | | | | | | |
| At 1 month |  | | | 11 ± 21 | |  | | | | 10 ± 23 |  | | | 0.09 |
| At 12 months |  | | | 12 ± 22 | |  | | | | 11 ± 22 |  | | | 0.05 |
| 1 month after end of treatment |  | | | 11 ± 21 | |  | | | | 11 ± 22 |  | | | 0.89 |
| Ventricular pauses | | | | | | | | | | | | | | |
| First week |  | | |  | | |  | |  | |  | | |  |
| ≥3 sec | 398 | | | 19 (4.8 %) | | | 379 | | 15 (4.0 %) | |  | | | 0.60 |
| ≥5 sec | 398 | | | 6 (1.5 %) | | | 379 | | 6 (1.6 %) | |  | | | >0.999 |
| At 30 days |  | | |  | | |  | |  | |  | | |  |
| ≥3 sec | 284 | | | 3 (1.1 %) | | | 255 | | 3 (1.2 %) | |  | | | >0.999 |
| ≥5 sec | 284 | | | 0 (0.0 %) | | | 255 | | 1 (0.4 %) | |  | | |  |
